# Cohort Profile: The Danish Testicular Cancer Late Treatment Effects Cohort (DaTeCa-LATE)

**DOI:** 10.3389/fonc.2018.00037

**Published:** 2018-02-21

**Authors:** Michael Kreiberg, Mikkel Bandak, Jakob Lauritsen, Julie Wang Skøtt, Nanna Borup Johansen, Mads Agerbaek, Niels Vilstrup Holm, Christoffer Johansen, Gedske Daugaard

**Affiliations:** ^1^Department of Oncology 5073, Copenhagen University Hospital, Rigshospitalet, Copenhagen, Denmark; ^2^Research Centre for Prevention and Health, Centre for Health, Glostrup, Denmark; ^3^Department of Oncology, Aarhus University Hospital, Aarhus, Denmark; ^4^Department of Oncology, Odense University Hospital, Odense, Denmark; ^5^Unit of Survivorship, Danish Cancer Society Research Center, Danish Cancer Society, Copenhagen, Denmark

**Keywords:** testicular cancer, germ cell cancer, cohort profile, cohort study, cancer late effects, late effects

## Abstract

The cohort was set up in order to analyze late effects in long-term testicular cancer survivors (TCS) and to contribute to the design of future follow-up programs addressing and potentially preventing late effects. Data for this cross-sectional study were collected between January 1, 2014, and December 31, 2016, among living Danish TCS and 60% agreed to participate in the cohort (*N* = 2,572). Mean time since testicular cancer (TC) diagnosis was 18 years (range 7–33) and mean age of participants was 53 years (range 25–95). Data consist of results of a questionnaire with patient reported outcomes which covers a broad range of items on late-effects. The study also included data obtained through linkages to Danish registries, a biobank, and clinical data from hospital files and pathology reports originating from the Danish Testicular Cancer Database (DaTeCa). The treatment during the observation period has been nearly the same for all stages of TC and is in agreement with today’s standard treatment, this allows for interesting analysis with a wide timespan. We have extensive data on non-responders and are able to validate our study findings. Data from a Danish reference population (*N* = 162,283) allow us to compare our findings with a Danish background population. The cohort can easily be extended to access more outcomes, or include new TCS. A limitation of the present study is the cross-sectional design and despite the large sample size, The Danish Testicular Cancer Late Treatment Effects Cohort (DaTeCa-LATE) lacks statistical power to study very rare late effects. Since it was voluntary to participate in the study we have some selection bias, for instance, we lack responders who were not in a paired relationship, but we would still argue that this cohort of TCSs is representative for TCSs in Denmark.

**Collaboration and data access:**

Researches interested in collaboration with the DaTeCa-LATE study group please contact Professor Gedske Daugaard kirsten.gedske.daugaard@regionh.dk.

## Introduction—Why was the Cohort Set Up?

In industrialized countries, testicular cancer (TC) is the most common solid tumor in men between 20 and 40 years of age. In Denmark, the age standardized incidence rate per 100,000 person-years is 9.9 which corresponds to 300 incident cases annually (1, 2). TC is a highly curable tumor with a 5-year survival of 95–98%, which results in an increased population of long-term testicular cancer survivors (TCS). However, treatment is hampered by late effects, including increased risk for secondary cancer (3, 4), metabolic syndrome (5), cardiovascular disease (6), neurotoxicity (7), sexual dysfunction (8), and psychosocial problems (9). Nevertheless, our current knowledge of risk factors, the related health problems and the quality of the posttreatment life remains insufficient to fully optimize individual programs designed to address and reduce long-term toxicity and improve quality of life in TC survivors.

Historically there have been small variations in standard TC treatment in Denmark, and now it is completely harmonized in national multidisciplinary guidelines for treatment and follow-up carried out at three university hospitals responsible for this patient group. The treatment algorithm is as follows: (a) patients with disease confined to one or both testicles (stage I disease) are treated with orchiectomy followed by surveillance, and approximately 75% of stage I patients are cured by this treatment alone (10, 11); (b) patients with seminoma histology and limited retroperitoneal disease are offered treatment with radiotherapy (RT) or chemotherapy; (c) in the remaining patients with disseminated disease, chemotherapy with three or four courses of bleomycin, etoposide, and cisplatin (BEP) is the treatment of choice depending on prognostic group; and (d) a minority of patients, approximately 5%, will need more than one line of treatment (MTOL) due to progressive disease or disease relapse.

In order to clarify late effects related to treatment, apart from orchiectomy, we have chosen, where appropriate, to use the surveillance group (stage I TC) as a reference group.

**Figure 1:**
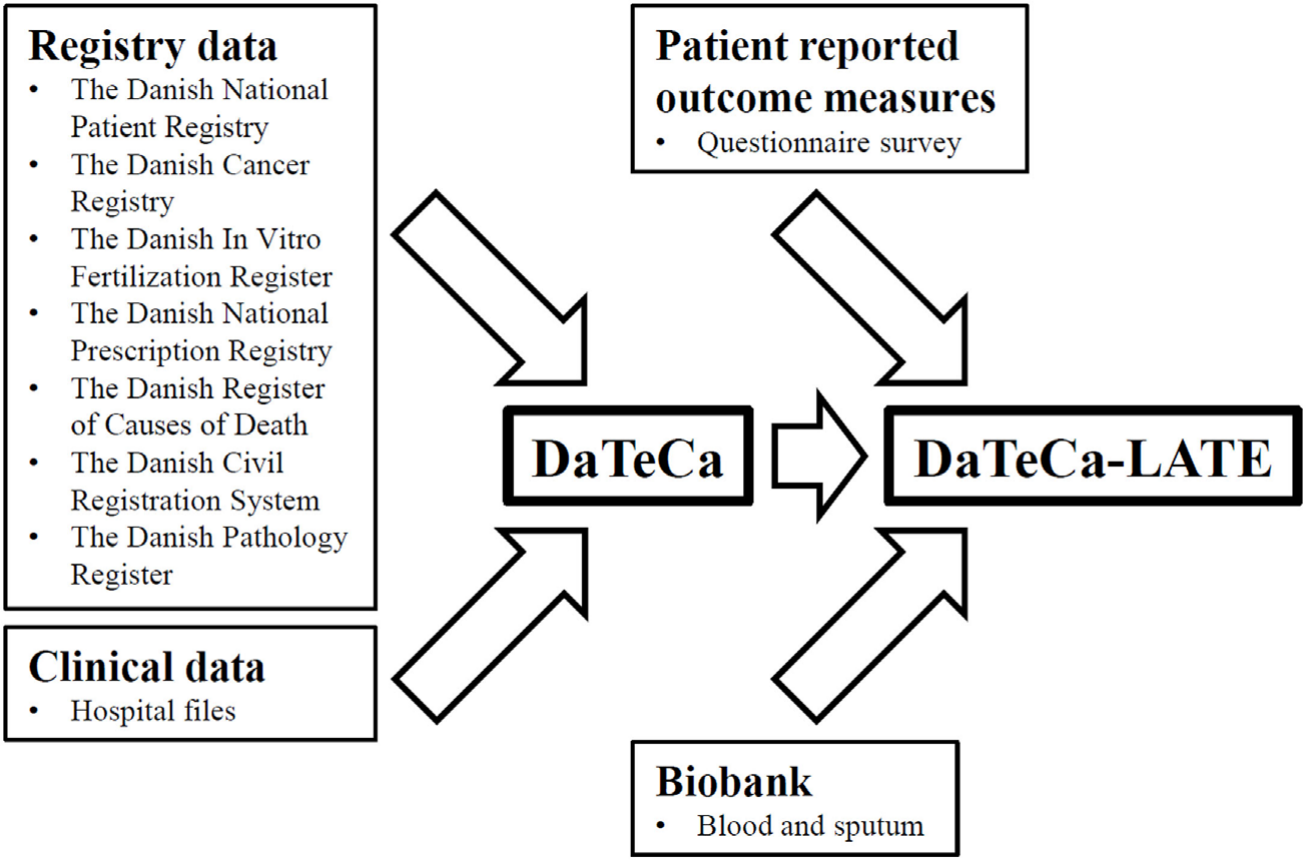
The Danish Testicular Cancer Late Treatment Effects Cohort (DaTeCa-LATE) cohort design.

The Danish Testicular Cancer Late Treatment Effects Cohort (DaTeCa-LATE) consists of a combination of data derived from the clinical database DaTeCa (1984–2007) (11), national health and sociodemographic registries (1977–2016), a biobank with blood and sputum samples, and an 167-item questionnaire containing patient reported outcome measures (PROMs) obtained in 2014–2016. The cohort design is illustrated in Figure 1.

The cohort was set up in order to analyze treatment-related late effects in TCS and to contribute to the design of future follow-up programs addressing and potentially preventing late effects in long-term TCS.

The Danish Testicular Cancer Late Treatment Effects Cohort is a nationwide and population-based cohort and is located at the Department of Oncology, Rigshospitalet, Copenhagen University Hospital, Denmark.

## Who is in the Cohort?

**Figure 2:**
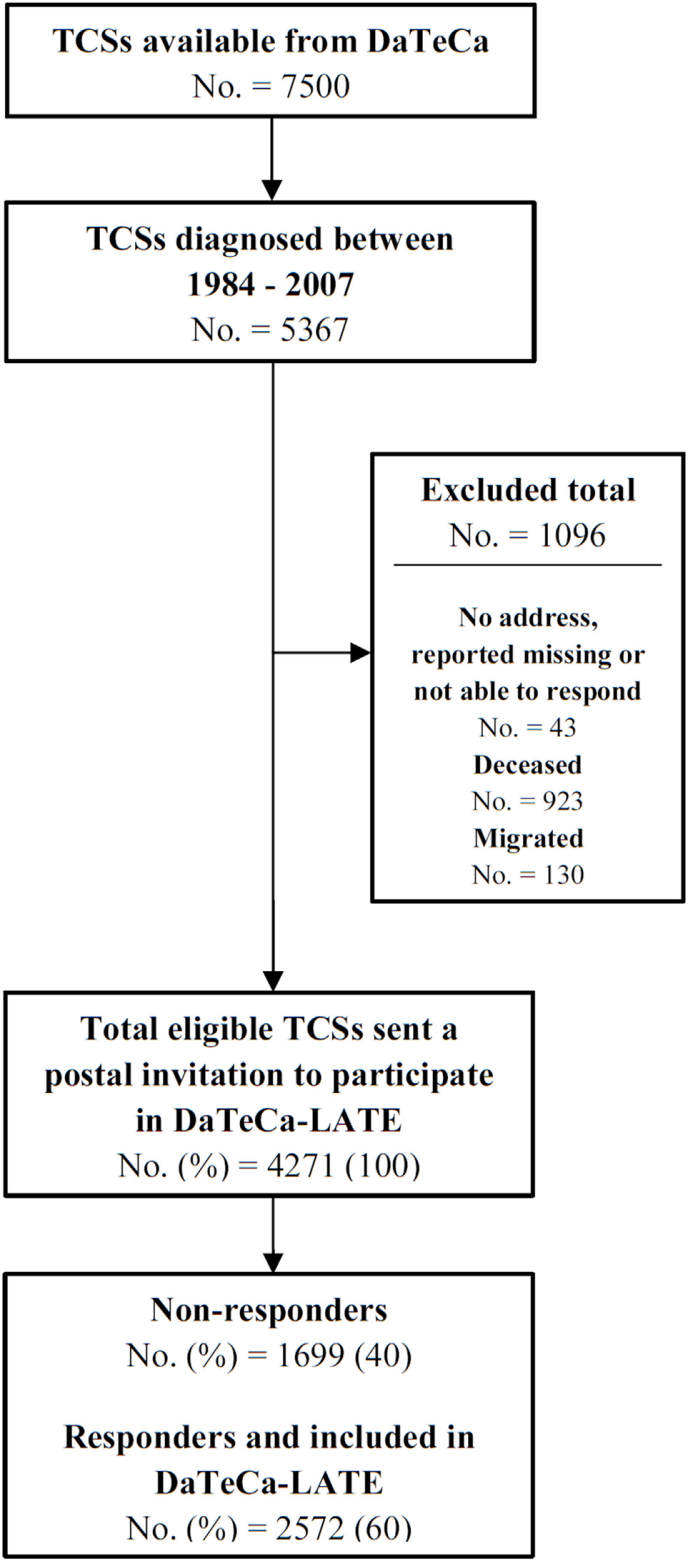
The Danish Testicular Cancer Late Treatment Effects Cohort (DaTeCa-LATE) flowchart.

In 2014, we accessed the DaTeCa database, which at that time included some 7,500 TC patients. Patients in the database have been identified through the Danish Cancer Register and hospital files. In the present study, we included 5,367 of these patients treated in the period between January 1, 1984, and December 31, 2007, as we aimed for a long observational period facilitating the study of late effects. The mean time since diagnosis is 18 years (range 7–33). Inclusion criteria in the DaTeCa database covers the diagnosis of a germ cell cancer (International Classification of Diseases, 10th revision, ICD-10: DC 62.1-62.9, DC38.3, and DC48.0, the latter two together with germ cell histology), Danish citizenship, follow-up and medical treatment conducted at an oncology ward in Denmark. The overall coverage in the database is approximately 80% (11). In the DaTeCa database, we identified a total of 4,271 TCS eligible for participation in DaTeCa-LATE. The main reasons for exclusion of patients (*N* = 1,096) are presented in the flowchart (Figure 2).

A postal invitation to participate was sent out to all eligible TCS in November 2014. Following written informed consent, patients filled in a questionnaire either in a paper version or electronically. Reminder postal invitations were sent out to non-responders and non-responders were also reached by phone. By December 31, 2016, a total of 2,572 (60%) of questionnaires distributed was filled in and returned, and this number of TCS make up the DaTeCa-LATE study population.

Data on TCS non-responders were collected from the DaTeCa database and Danish registries for comparison with responders. As illustrated in Table 1 responders were younger at time of treatment (34 vs. 35 years) and attained age (52 vs. 53 years) than non-responders (*P* < 0.001).

**Table 1:** Characteristics of responders included in DaTeCa-LATE and non-responders.

Characteristics	Responders, no. (%) 2,572 (60)	Non-responders, no. (%) 1,699 (40)	*P*-Value
Age treatment (years)			<0.001
Mean	35	34	
SD	10	9	
Age attained (years)			<0.001
Mean	53	52	
SD	11	11	
Time since diagnosis (years)			0.014
Mean	18	17	
SD	7	7	
Decade of treatment, no. (%)			0.051
1980s	514 (20)	289 (17)	
1990s	1,126 (44)	773 (46)	
2000s	932 (36)	637 (37)	
Treatment, no. (%)			<0.001
Surveillance	1,174 (46)	947 (56)	
BEP	896 (35)	441 (26)	
RT	323 (12)	236 (14)	
MTOL	85 (3)	27 (1)	
Other^a^	94 (4)	48 (3)	
Histology, no. (%)^b^			0.384
Seminoma	1,370 (53)	935 (55)	
Nonseminoma	1,201 (47)	764 (45)	
Comorbidity, no. (%)			0.190
No comorbidity	2,207 (86)	1,424 (84)	
One or more comorbidities	365 (14)	275 (16)	
Place of living, no. (%)			0.444
Urban	857 (33)	547 (32)	
Rural	1,715 (67)	1,152 (68)	
Marital status, no. (%)			<0.001
Paired relation	1,697 (66)	911 (54)	
Single, separated, divorced, widower	875 (34)	788 (46)	

*Age treatment, age attained. and time since diagnosis were compared with independent t-test. Column proportions were compared through chi-squared analysis*.

*^a^Metachronous and synchronous*.

*^b^Extragonadal primary (*N* = 123)*.

*BEP, bleomycin, etoposide, cisplatin; RT, radiotherapy; MTOL, more than one line of treatment*.

The response rate was dependent on treatment modality. As such, the response rate was 55% in TCS followed on a surveillance program, 67% in TCS treated with BEP, 58% in TCS treated with RT, and 76% in TCS treated with MTOL. These data suggest that more intensive treatment lead to a higher response rate. Another difference observed, concerns marital status. TCS living in a paired relation had a response rate of 66% compared to men who were single, separated, divorced, or widowed with a response rate of 54% (*P* < 0.001). No difference between responders and non-responders were observed concerning decade of treatment, histology of the tumor, comorbidity at time of filling in the questionnaire and place of living.

As a reference population we use data obtained from the Danish National Health Profile survey from 2013 based on five regional stratified random samples and one national random sample. A total of 300 450 individuals, aged 16 or older, were invited to participate with a response rate of 54% (162,283 individuals) (12). As many of the outcomes in the Danish National Health Profile survey are the same as in DaTeCa-LATE this allows for a straight-forward comparison with the reference population, concerning the following demographic variables and outcomes: alcohol consumption, smoking, education, age, self-rated health, physical activity, perceived stress, physical function, and body mass index.

In the present study, continuous variables were compared with independent *t*-tests while column proportions were compared with chi-squared analysis. Statistical analyses were performed by using SPSS 22.0 (SPSS, Chicago, IL, USA).

The regional ethical committee of the Capital Region of Denmark approved the study (file number, H-2-2012-044).

The collection and use of questionnaire data from the reference population were approved by the five regional data protection agencies.

This report covers cross-sectional data obtained at December 31, 2016, only.

## What has been Measured?

Variables in DaTeCa-LATE were chosen based on literature describing late effects in TCS together with clinical experiences concerning late effects in cancer patients. Table 2 gives an overview on variables in DaTeCa-LATE in addition to the variables originating from the DaTeCa database.

**Table 2:** Outcomes measured in DaTeCa and DaTeCa-LATE surveys.

Sources		Outcomes
**DaTeCa**		
Clinical data		
1984–2015	Hospital files	Stage (prognostic group, metastatic site), treatment, relapses, kidney function, lung function, pathology reports, ototoxicity, neurotoxicity, smoking at diagnosis

Registry data		
1977–	The Danish National Patient Registry	Comorbidity^a^
1984–	The Danish Cancer Registry	Diagnosis, histology
1993–	The Danish *In Vitro* Fertilization Register	Help to achieve pregnancy with spouse
1995–	The Danish National Prescription Registry	Use of medication
1984–	The Danish Register of Causes of Death	Cause of death
2014	The Danish Civil Registration System	Marital status, place of living, birthplace, migration
1984–	The Danish Pathology Register	Tumor characteristics

**DaTeCa-LATE**		

PROM		
2014–2016	Fatigue	Multiple fatigue inventory (MFI-20)
	Quality of life	EORTC QLQ-C30
	Anxiety and depression	Hospital anxiety and depression scale (HADS)
	Psychological distress	Perceived stress scale (PSS)
	Demographics	Education level, occupation, income
	Alcohol and tobacco	Type, duration and frequency
	Substance abuse history	Amphetamines, cocaine, heroin, hallucinogens, marijuana, barbiturates, ecstasy, steroids
	Health	Self-rated, behaviors. Type and frequency in use of medication. Height, weight
	Exercise	Type, frequency, self-rated physical health
	Family history	Medical diagnoses in first- and second-degree relatives
	Pain	Localization, intensity
	Neurotoxicity	FACT/GOG-NTX subscale
	Infertility before and after testicular cancer diagnosis	Use of sperm bank, children, type and use of medical help
	Symptoms of testosterone deficiency and erectile dysfunction	International Index of Erectile Dysfunction (IIEF-15)
	Medical history and symptoms	Use of medication, diseases in relatives, respiratory symptoms

Biobank		

2014–2016	Blood and sputum	Future genetic analyses

*^a^Since 1993, where all data are registered according to ICD10*.

*PROM, patient reported outcome measures*.

## Hospital Files and Pathology Reports

Clinical data in DaTeCa-LATE are obtained from hospital files and pathology reports and are available from the DaTeCa database (11), and depending on stage and treatment, more than 300 variables are available covering histology, stage, tumor markers, treatment, relapses, kidney function, and lung function.

## Danish Registries

Since April 1968, every Danish resident has been assigned a unique civil personal 10-digit registration number. Each individual is identified with this number and it can be linked to different health-related and sociodemographic registries.

Seven different Danish national registries have for now contributed to DaTeCa-LATE. From the Civil Registration System (13), we retrieved the dates of migration and cohabitation status, information on comorbidity were collected using The National Patient Registry (14), and The Danish Cancer Registry provided information on cancer diagnosis in order to identify the patients (15). Combining hospital files including pathology reports and data from the Danish Pathology Register data on tumor characteristics were obtained (16).

The Danish *in Vitro* Fertilization Register provided information on help to achieve pregnancy (17), from the Danish National Prescription Registry knowledge of prescribed medications have been obtained (18), and by linkage to the Registry of Causes of Death we obtained the dates and causes of death (19).

## Patient Reported Outcome Measures (PROM)

The abovementioned questionnaire contain PROMs concerning quality of life (EORTC QLQ-30) (20), fatigue (Multiple Fatigue Inventory) (21), symptoms of testosterone deficiency and erectile dysfunction (International Index of Erectile Dysfunction) (22), psychological distress [perceived stress scale (PSS)] (23), depression and anxiety (hospital anxiety and depression scale) (24), and neurotoxicity (FACT/GOG-NTX) (25). Additional PROM outcomes are education, occupation, income, alcohol, tobacco, drug use and abuse, self-rated health, physical activity, anthropometrics, pain, and infertility.

## Biobank

A total of 430 TC patients treated with chemotherapy have contributed with DNA samples derived from sputum or blood in order to identify predictive genetic markers for long-term toxicities. Furthermore, in 2012, additionally 245 TC patients had provided serum, plasma and DNA samples for the Danish Cancer Biobank (26), and since then nearly all TC patients have provided samples for later analyses.

## Previous, Ongoing, and Planned Studies

We have previously reported that patients followed on surveillance are at no higher risk for a new primary cancer compared to the background population (3), while this is the case for patients treated with chemotherapy, RT, or MTOL for disseminated disease, who also have increased mortality compared to patients with stage I disease (3, 27). Furthermore, we have found that renal and pulmonary toxicity related to treatment is partly reversible (28, 29). Risk of late relapses is low in surveillance patients, who carries a good prognosis (30, 31). Additionally, we have shown that patients with preexisting Leydig cell dysfunction are at increased risk of testosterone deficiency following treatment (32–34).

**Figure 3:**
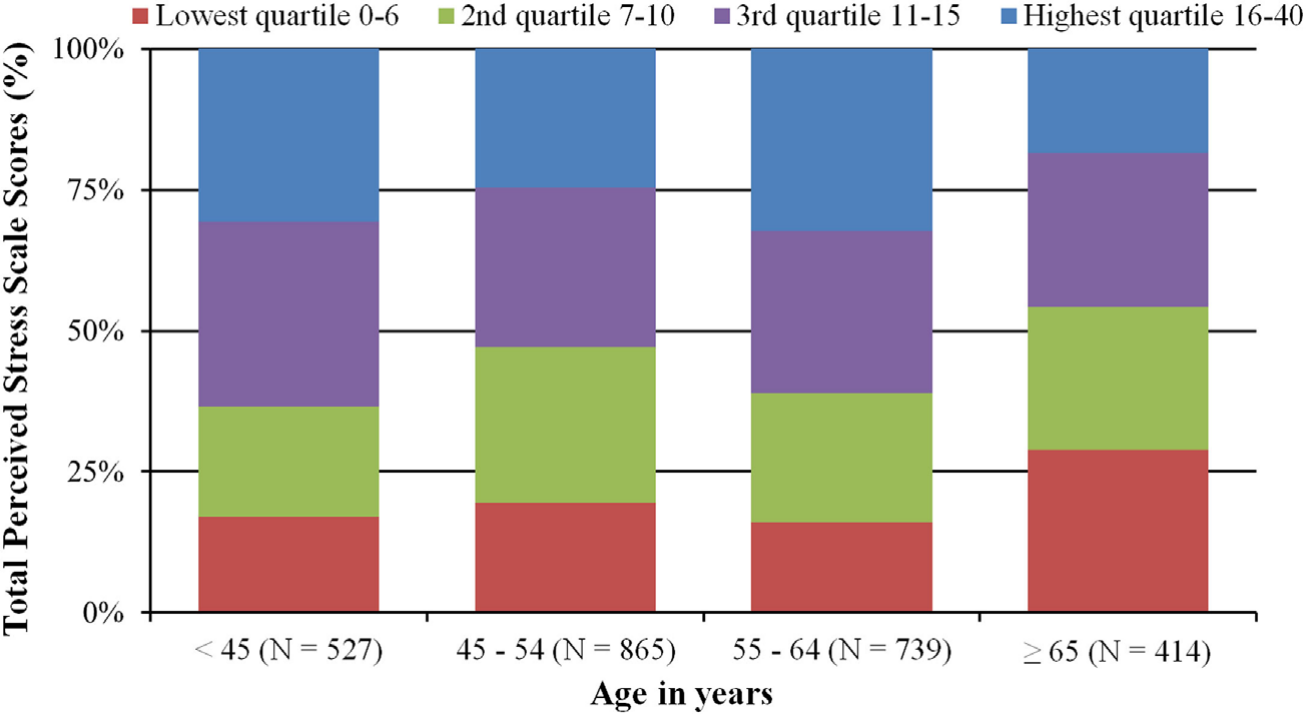
Testicular cancer survivors perceived stress scale (PSS) scores categorized in age groups and allocated into quartiles according to reference group quartile PSS scores.

A new finding from DaTeCa-LATE is that TCS have higher PSS scores than the reference population. This is illustrated in Table 3, where mean PSS scores were compared between TCS and the reference population with independent *t*-test. No cutoff values exist for PSS, but generally a score of 15 or above is considered as a high stress level (35). In Figure 3, TCSs PSS score was categorized in age groups and allocated into quartiles according to reference group quartile PSS scores. Confined to TCS < 65 years of age we found a higher level of perceived stress compared to the reference population. In total, 16–19% of TCS < 65 years of age had PSS scores in the lowest quartile, while 53–64% had PSS scores in the two highest quartiles.

**Table 3:** Perceived stress scale (PSS), total scores.

	Testicular cancer survivors	Reference population	*P*-Value
PSS mean total score	11.6	10.7	<0.001
SD	6	7	
No.	2,545	69,438	

*PSS mean total score compared with independent t-test*.

To further elaborate on this primary analysis of PSS, we plan comparison of TCS level of perceived stress and lifestyle factors (physical activity, smoking, and alcohol consumption) with the reference population. In addition we will compare PSS scores of TCS with survivors of other cancer types.

Planned studies from DaTeCa-LATE include evaluation of quality of life and sexual function in bilateral TC as well as analysis of quality of life, education, psychiatric diseases and drug abuse, fatigue, sexual function, and paternity rate stratified on treatment modality and compared with a non-cancerous reference population when applicable. In addition, genetic differences related to toxicity and more rarely explored late-effects will be investigated.

Statistical analyses will be performed using SPSS (SPSS, Chicago, IL, USA) and R software (R Core Development Team, Vienna, Austria).

We plan an additional follow-up survey of the cohort after 5–10 years as well as an extension adding more recently diagnosed cases of TC.

## What are the Main Strengths and Weaknesses?

The Danish Testicular Cancer Late Treatment Effects Cohort is the largest cohort of TCS with a long observation period since diagnosis and treatment. The study is nationwide and population based which together with the long time since diagnosis leads to a high degree of reliability of the results obtained in various studies. The study includes a combination of an excessive number of clinical data, comprehensive PROM data, and access to various administrative register data established independently of the hypothesis under study. This type of data almost completely avoid the possibility that selection, information as well as recall bias influence the results, securing a high validity of the observations.

The treatment during the observation period has been nearly the same for all stages of TC and is in agreement with today’s standard treatment. Patients responding to the questionnaire can be compared with non-responders and a reference population. The cohort can easily be extended to assess additional outcomes, or include new TC patients.

A possible limitation is the differences observed in age, marital status and treatment modality between responders and non-responders of the questionnaire. However, the absolute difference in age is negligible and the proportion of patients treated with surveillance, BEP and RT is representative of today’s TC treatment pattern. Thus, we do not expect that these differences will influence the interpretation of PROM data. The difference in marital status with more responders than non-responders living in a paired relation is in accordance with the findings of other similar studies (36), and the finding can be problematic in special cases, but overall we find that this cohort of TCSs is representative for TCSs in Denmark.

As >95% of TC patients become long-term survivors, survivor bias is generally not a limitation. However, as the mortality is high in the MTOL group, interpretation of especially PROM data among these patients should be done with caution, as they might represent a highly selected group of TC who have survived several lines of TC treatment. Despite the fact that TC is a rare tumor implying that some rare outcomes would be difficult to investigate, the advantages of the information in this large cohort of TC provide a data set that is highly interesting for future studies.

## Can I Get Hold of the Data? Where Can I Find Out More?

The DaTeCa-LATE steering group welcomes collaboration and the interest of national and international colleagues. External researchers can get access to data by a collaboration agreement with the DaTeCa-LATE steering group. For more information on how to apply, please contact Professor Gedske Daugaard, kirsten.gedske.daugaard@regionh.dk.

## Ethics Statement

The regional ethical committee of the Capital Region of Denmark approved the study (file number, H-2-2012-044). The study was carried out in accordance with their recommendations. All subjects gave written informed consent in accordance with the Declaration of Helsinki.

## Author Contributions

MK, MB, JL, CJ, and GD contributed to data collection, data analyses, and drafting the manuscript. All authors contributed to the writing of this manuscript. MK, MB, JL, JS, NJ, MA, NH, and GD contributed to data collection and data quality. All authors read and approved the final manuscript.

## Conflict of Interest Statement

The authors declare that the research was conducted in the absence of any commercial or financial relationships that could be construed as a potential conflict of interest.
